# Targeting the chemokines in acute graft-versus-host disease

**DOI:** 10.3389/fimmu.2024.1525244

**Published:** 2025-01-07

**Authors:** Ziwei Xu, Huafang Wang

**Affiliations:** Institute of Hematology, Union Hospital, Tongji Medical College, Huazhong University of Science and Technology, Wuhan, China

**Keywords:** chemokines, acute graft-versus-host disease, allogeneic hematopoietic stem cell transplantation, T cells, diagnostic biomarkers

## Abstract

Allogeneic hematopoietic stem cell transplantation (allo-HSCT) constitutes a critical therapeutic approach for patients with malignant hematological disorders. Nevertheless, acute graft-versus-host disease (GVHD), one of the most prevalent complications associated with HSCT, remains a leading contributor to non-relapse mortality. In recent years, there has been an increasing focus on the interplay between chemokines and their receptors in the context of acute GVHD. Chemokines exert substantial effects across various pathological conditions, including autoimmune diseases, inflammatory processes, tumorigenesis, and metastatic dissemination. In this review, we aim to elucidate the role of chemokines in the pathogenesis of acute GVHD and further understand their potential as diagnostic biomarkers. We also present both preclinical and clinical insights into the application of chemokines in preventing and treating acute GVHD. The objective of this review is to offer novel perspectives on the clinical diagnosis and management strategies for acute GVHD.

## 1 Introduction

Allogeneic hematopoietic stem cell transplantation (allo-HSCT) is a cornerstone of therapy for patients with malignant hematological disorders that is intended to achieve enduring remission (1). However, allo-HSCT is associated with several complications, including most notably graft-versus-host disease (GVHD). Despite continuous advancements in pharmaceutical formulations and preventive strategies intended to alleviate acute GVHD, grade II-IV acute GVHD following transplantation is observed in 40-50% of patients and is associated with poor long-term prognosis and reduced survival (2, 3). The underlying discordance in tissue compatibility between donor and recipient triggers donor T cells to misidentify host tissues as foreign, thereby precipitating the onset of acute GVHD (4). The intricate pathophysiology of acute GVHD unfolds in three sequential phases: initiation phase, T cell activation, and effector phase (3).

Chemokines, which are small molecules of around 8-14 kDa secreted by various cell types, serve as pivotal orchestrators in numerous biological processes. They are systematically categorized into four subfamilies—: CCL, CXCL, XCL, and CX3CL—based on their conserved cysteine residues (5). The human spectrum encompasses approximately 50 chemokines, which facilitate precise cellular migration by recognizing seven transmembrane chemokine receptors (CCR, CXCR, XCR, and CX3CR) linked to G proteins on the cell surface (6). These versatile molecules exert profound influences across autoimmune conditions, inflammatory cascades, tumorigenesis, and metastatic dissemination (7–10). By binding to specific receptors on immune cells, chemokines are directed to sites of inflammation, where they participate in the activation, differentiation, and effector functions of immune cells (11). Given their central position in immune regulation, chemokines have emerged as important players in the development and progression of acute GVHD. In the context of acute GVHD, activated allogeneic donor CD4^+^ and CD8^+^ T cells are adeptly guided to their targets by chemokines, ultimately eliciting tissue damage via apoptosis and necrosis (12, 13).

In this review, we provide insight into the mechanisms underpinning chemokine modulation of the immune response during acute GVHD, as well as a comprehensive description of the pivotal roles of chemokines in the pathophysiology of acute GVHD and an overview of novel strategies that are currently under investigation.

## 2 The pathophysiology of acute GVHD

### 2.1 Initiation phase

To achieve optimal eradication of the recipient’s hematopoietic system, tumor cells, and immune components, a high-intensity, myeloablative conditioning regimen is the preferred approach for most patients. The administration of high-dose, myeloablative total body irradiation (TBI) and cytotoxic agents renders patients highly vulnerable to disturbances by the gut microbiota, resulting in upregulated expression of cytokines such as tumor necrosis factor-α (TNF-α), interleukin-1β (IL-1β), and interleukin-6 (IL-6), which can induce inflammation and tissue damage (14–16). Pathogen-associated molecular patterns (PAMPs), consisting of structurally conserved molecular features prevalent on the surfaces of diverse pathogenic microorganisms, and damage-associated molecular patterns (DAMPs), which are endogenous molecules originating from cellular damage within the host, are two classes of molecules that play pivotal roles in the initiation phase (16, 17). Conditioning or infection-induced tissue injury facilitate the translocation of PAMPs into the bloodstream or lymphatic tissues, simultaneously triggering the release of DAMPs. Upon engagement with pattern recognition receptors (PRRs), these small molecules promote the activation of allogeneic T cells (17). Notably, PAMPs such as lipopolysaccharide (LPS) and nucleotide-binding oligomerization domain containing 2 (NOD2) have been implicated in the pathogenesis of acute GVHD (18, 19). PAMPs derived from the gut microbiota recognize and interact with toll-like receptors (TLRs), thereby stimulating myeloid cells and epithelial cells to secrete pro-inflammatory cytokines, exacerbating acute GVHD (20). Additionally, DAMPs, including uric acid, adenosine triphosphate (ATP), and interleukin-33 (IL-33), released from damaged tissues such as the intestinal epithelium, activate T cells, and antigen-presenting cells (APCs), ultimately culminating in an inflammatory responses (21).

### 2.2 T cell activation

Chemokines not only facilitate T-cell migration but also potentially enhance their infiltration and activation within target organs. Once activated by PAMPs and DAMPs, both classic APCs such as B cells, dendritic cells (DCs), and macrophages, as well as non-classic APCs such as basophils and mast cells, promote the activation of donor T cells (22). These APCs process and present both major and minor human leukocyte antigens (HLA), triggering the initiation, activation, and proliferation of donor T cells. Subsequently, activated T cells release cytokines, including interleukin-2 (IL-2), interleukin-15 (IL-15), and interferon-γ (IFN- γ) (23). Guided by chemokines, activated T cells traverse the vascular endothelium and migrate towards target organs. These chemokines promote not only T cell migration but also potentially enhance their infiltration and activation within target organs (24).

### 2.3 Effector phase

**Figure 1:**
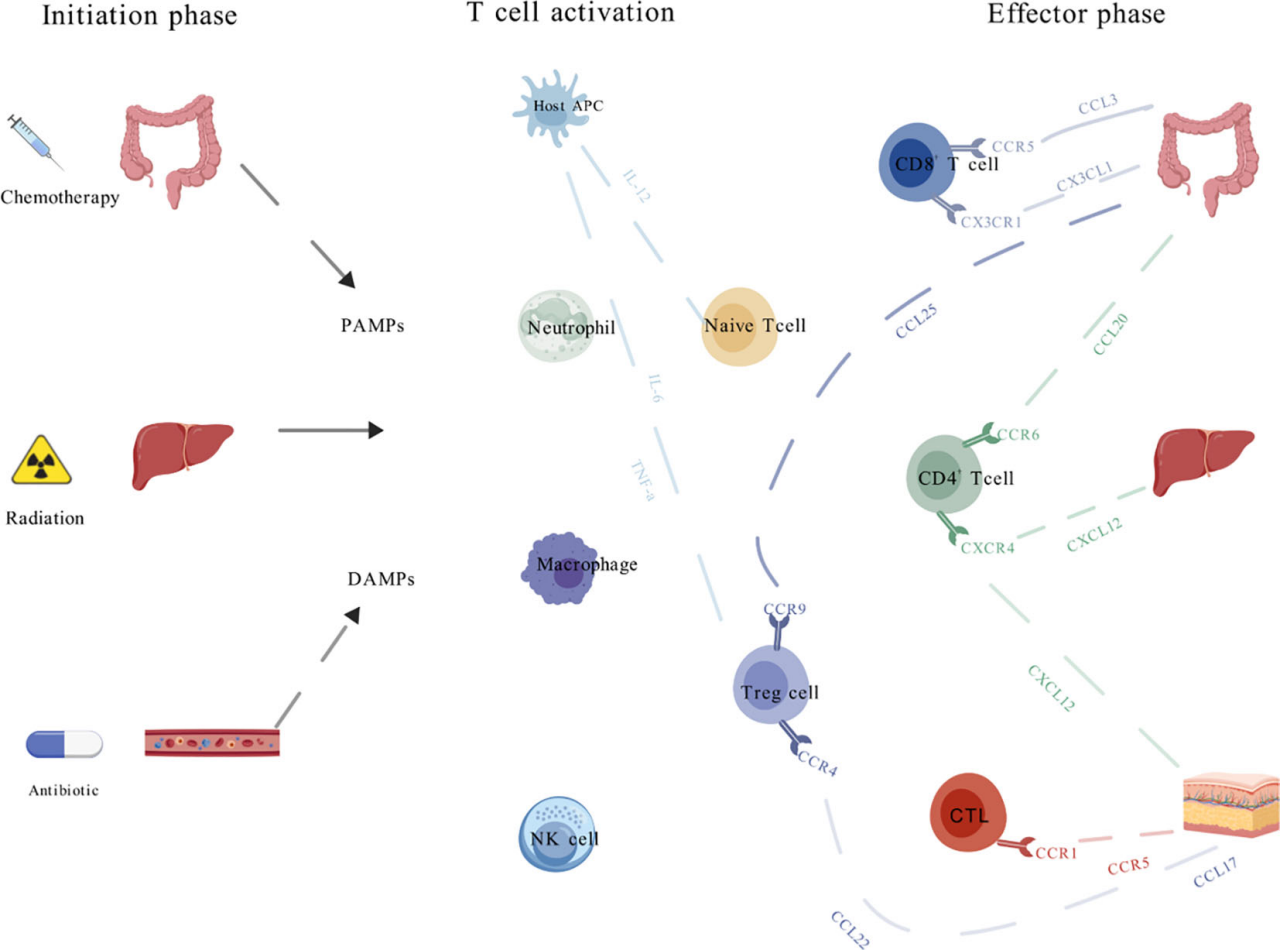
Role of chemokines in the development of acute graft-versus-host-disease. The pathophysiology of acute graft-versus-host disease (GVHD) is characterized by three sequential stages. In the initiation phase, tissue damage resulting from conditioning regimens such as chemotherapy or radiation disrupts the integrity of intestinal epithelium, leading to the release of pathogen-associated molecular patterns (PAMPs) and damage-associated molecular patterns (DAMPs). This is followed by the activation of donor T cells, wherein naïve T cells differentiate into various subtypes and undergo clonal expansion. During the effector phase, activated T cells migrate to target organs through interactions between surface receptors and chemokines, culminating in tissue injury.

During the effector phase, a positive feedback loop mechanism, driven by the continuous recruitment of immune cells, significantly aggravates tissue damage (**Figure 1**). Cytokines play a pivotal role at this point, orchestrating the differentiation of CD4^+^ and CD8^+^ T cells into cytotoxic T cell (Tc cells) subsets (25). These Tc cells induce apoptosis in target cells through distinct pathways: the Fas/Fas ligand signaling cascade and the perforin-granzyme-mediated mechanism, thereby fulfilling their immunological effector functions (26, 27). Recent studies have revealed that by meticulously modulating the expression and function of chemokines, it is possible to prevent the targeted migration and excessive recruitment of T cells towards affected tissues (28). Additionally, this modulation can fine-tune the differentiation balance among T-cell subsets, ultimately mitigating the severity of acute GVHD. This approach provides a promising avenue for the development of novel therapeutic strategies against acute GVHD.

## 3 The roles of chemokines in acute GVHD

The coordinated movement of T cells throughout the body in response to specific stimuli is a fundamental factor in the immune response. This process is intricately regulated by chemokines, which control the directional movement of immune cells. Chemokine-mediated T-cell trafficking involves a complex interplay between adhesion molecules, chemokines, and their receptors. The process of T-cell trafficking commences with the recognition of a chemokine gradient by chemokine receptors expressed on the surface of T cells. Upon binding to their respective receptors, chemokines activate integrins, which are adhesion molecules that mediate cell-cell and cell-matrix interactions. This activation results in increased adhesion between T cells and endothelial cells lining the blood vessels, enabling T cells to extravasate into the surrounding tissues (29). For T-cell trafficking, CC and CXC chemokines are particularly important, with CC chemokines primarily involved in the recruitment of monocytes, eosinophils, basophils, and T-helper (Th) cells, while CXC chemokines attract neutrophils, some monocytes, and Tc cells (7). In this section, we will explore the mechanisms underlying chemokine-mediated T-cell trafficking, highlighting the key players, interactions, and signaling pathways involved.

### 3.1 CCR1

In xenograft mouse model, CCR1 mRNA was upregulated in the liver and intestines suggesting that CCR1 may play a vital role in the pathogenesis of acute GVHD (30). Further studies showed that recipient mice receiving CCR1 ^−/−^ donor cells exhibited significantly reduced severity of acute GVHD compared to the wild type (WT) donor cell group (30). Importantly, this improvement was accompanied by a significant decrease in the infiltration of mononuclear cells and neutrophils in the intestine, further validating the critical role of CCR1 in regulating the migration of immune cells to inflammatory sites. Additionally, CCL5, as one of the main ligands of CCR1, plays an indispensable role in the recruitment of antigen-specific activated Th cells and Tc cells to inflammatory tissues, thereby mediating the process (31, 32).

### 3.2 CCR2

Monocyte chemoattractant protein-1 (MCP-1, also known as CCL2), plays a crucial role in the chemotaxis of monocytes and T cells (33, 34). In the acute GVHD mouse model, activated CD8^+^ T cells highly express CCR2 and migrate under the guidance of CCL2 (35). This phenomenon is absent in CCR2-deficient mice, accompanied by a significant reduction in the infiltration of CD8^+^ T cells into the liver and intestines and a decrease in the severity of tissue damage. Additionally, CCL2/CCR2 also mediates the migration of activated macrophages to the mucosal surface, closely related to the tissue destruction observed in oral acute GVHD (36).

### 3.3 CCR4

A comparison of skin tissue biopsies from patients with acute GVHD vs those without revealed a significant increase in the proportion of CD70^+^ subgroups within CD8^+^ T cells, surpassing the increase observed in CD70^+^ subgroups within CD4^+^ T cells (37). This phenomenon may be attributed to the expression levels of chemokine receptors, particularly CCR4 and CCR6, on CD70^+^ T cells, which are significantly higher in acute GVHD patient tissues than in peripheral blood, with particularly pronounced upregulation of CCR4 (37). As a major chemokine receptor expressed on T cells, CCR4 specifically binds to the CC chemokine ligands CCL17 and CCL22, warranting further exploration of its role in GVHD pathogenesis (38). Mogamulizumab, a humanized anti-CCR4 monoclonal antibody, has been approved for the treatment of adult T-cell leukemia/lymphoma (ATLL) (39). However, studies have indicated a remarkable increase in the risk of acute GVHD and non-relapse mortality in patients treated with Mogamulizumab prior to HSCT (40, 41). This may be related to the activity of Mogamulizumab depleting Tregs that express high levels of CCR4 (42, 43). For ATLL patients undergoing HSCT, the use of Mogamulizumab may pose additional risks for the development of acute GVHD.

### 3.4 CCR5

CCR5 can interact with a plethora of ligands, including CCL3, CCL4, and CCL5. Upon binding to its ligand, CCR5 orchestrates the migration and functional activities of lymphocytes, monocytes, and macrophages (44). During the inflammatory process, the binding of CCR5 and its ligand mediates the recruitment of effector molecules to the target organ, thereby regulating the ensuing activation that ultimately culminates in tissue damage. Given the extensive range of cell types that express CCR5, this chemokine is implicated in the pathophysiological mechanisms of numerous diseases, spanning energy metabolism, cellular senescence, apoptosis, infection, immunity, inflammation, angiogenesis, and tumorigenesis (45–51). Pathological analysis of patients with skin acute GVHD revealed high expression of CCR5 in CD4^+^ and CD8^+^ T cells (52). The levels of CCL3 secreted by bile duct epithelial cells and endothelial cells were significantly increased in the acute GVHD mouse model, which recruited CCR5^+^CD8^+^ T cells to the liver, leading to significant tissue damage (53). Moreover, CCR5 is crucial for the migration of Treg and the recruitment of CD8^+^ T cells to Peyer’s patches in the intestine, and the blockade of CCR5 not only reduces the number of donor-derived T cells and the ratio of Th1/Th17 subpopulations but also inhibits the maturation of DCs (28, 54, 55).

The extent of T cell proliferation is intricately linked to the phosphorylation levels of AKT, 4E-BP1, and RPS6. PI3K inhibitors target the PI3K/AKT/mTOR pathway to suppress T cell activation and mitigate acute GVHD organ damage (56). In a murine model of acute GVHD, blockade of PI3Kγ resulted in a reduction in the expression of pro-inflammatory chemokines, namely CCL3 and CCL5. Moreover, it attenuated leukocyte adhesion in the mesenteric microcirculation (57). PI3Kγ likely contributes to the pathogenesis of acute GVHD by regulating chemokine expression. *In vitro* experiments demonstrated that angiogenesis primarily initiates tissue inflammation prior to leukocyte infiltration in the acute GVHD (58). Vascular endothelial growth factor A (VEGF-A), which promotes endothelial cell migration, is associated with CCL5/CCR5 (59). Elevated levels of TNF-α and IFN-γ activate CCL5/CCR5, whereas p65 nuclear translocation promotes NF-κB signal transduction (60). The NF-κB signaling pathway plays a pivotal role in the alloresponse and represents a promising target for preventing acute GVHD (61). Additionally, CCL5 binding to the CCR5 stimulates the mTOR pathway, thereby promoting cellular growth. Consequently, the expression of cyclin D1 and c-Myc rapidly increases, activating the JAK/STAT pathway (62). The Ruxolitinib, a JAK1/JAK2 inhibitor, has been approved for the treatment of steroid-resistant acute GVHD and has demonstrated favorable therapeutic effects (63).

Notch signaling is implicated in the pathophysiology of acute GVHD, as it regulates T-cell activation and differentiation (64). Blocking the delta-like Notch ligand DLL4 protects against gastrointestinal GVHD and improves survival in nonhuman primate models (65). Notch signaling upregulates α4β7 integrin in T cells post-allo-HSCT, affecting the ratio of conventional T cells to Treg cells (65). Selective CCR5 antagonists exhibit potential neuroprotective effects in multiple sclerosis by downregulating NF-κB/Notch signaling (66). Notch signaling plays a vital role in regulating CCR5 and CCL5 expression and exerting biological effects in T cell acute lymphoblastic leukemia and breast cancer, respectively (67, 68). The onset of acute GVHD is delayed following the administration of MEK inhibitors, which decrease cytokine production by activated T cells (69). The phosphorylation of ERK1/2 in CD4^+^ T cells at day +30 is associated with acute GVHD patients, and its reduction corresponds to the alleviation of acute GVHD symptoms (70).

Activation of MAPK pathway drives significant increases in CCL5 secretion by tumor cells, thereby impacting the abundance of Treg cells (71). Conversely, the CCL5/CCR5 axis can also activate the Ras/MAPK pathways, leading to increased expression of proteins such as p38 and p-ERK1/2 (71).

**Figure 2:**
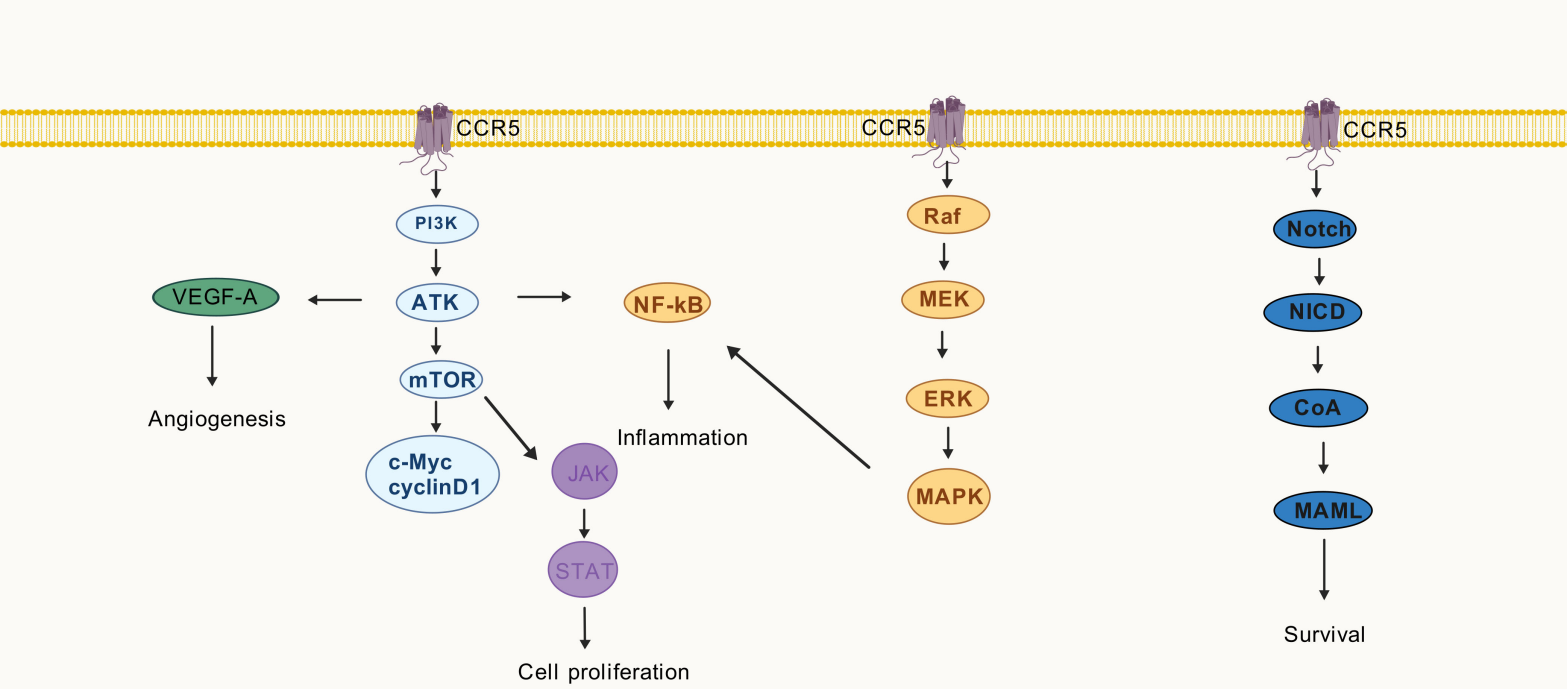
The CCR5 in different pathways associated with acute graft-versus-host disease.

Throughout the progression of acute GVHD, the CCR5 receptor binds to its respective chemokine and triggers relevant signaling pathways as showed in **Figure 2**. Some of these pathways enhance the secretion of pro-inflammatory chemokines, exacerbating the inflammatory response.

### 3.5 CCR6 and CCR7

CCR6/CCL20 assumes a crucial role in enrolling allogeneic reactive CD4^+^ T cells to target tissues, while CCR7, as a receptor for homeostatic chemokines, together with its ligands CCL19 and CCL21, maintains the efficient homing of T cell subpopulations to lymph nodes (72, 73). Deficiency in CCR7 expression can limit the expansion of Treg cells, thereby promoting the occurrence of acute GVHD (74).

### 3.6 CCR8

Recipient mice that received donor cells with CCR8 knocked out demonstrated a significant reduction in Treg cells in mesenteric lymph nodes and Peyer’s patches at day +10 post-transplantation compared to mice receiving WT donor cells (75). This mechanism may be related to the augmented apoptosis of Treg cells subsequent to CCR8 blockade, ultimately resulting in lethal acute GVHD in mice (75).

### 3.7 CCR9

Furthermore, CCR9 and its ligand CCL25 are highly expressed in the intestinal endothelial venules and Peyer’s patches and play a key role in the pathogenesis of acute GVHD (76, 77). The protective role of Treg cells in acute GVHD is well-known (78, 79), and donor Treg cells with CCR9 overexpressing can further alleviate the severity of acute GVHD and prolong the survival of mice (80). This mechanism may be associated with the promotion of Treg cells migration and accumulation in the intestine during the early stages of transplantation due to CCR9 overexpression, thereby inhibiting the secretion of inflammatory cytokines (such as TNF-α and IFN-γ) by CD4^+^ and CD8^+^ T cells and reducing intestinal tissue damage (80).

### 3.8 CCR10

In pediatric patients with skin acute GVHD, the proportion of CCR10^+^ CD4^+^ subgroups in peripheral blood T cells is significantly higher than in non-GVHD patients, and this subgroup vanishes after the resolution of acute GVHD (81). Additionally, skin tissue biopsies have revealed a close correlation between the infiltration of CCR10^+^ CD4^+^ T cells and the increased expression of the CCR10 ligand CCL27 (81). These findings suggest that the interaction between CCL27 and CCR10 may participate in the recruitment of CD4^+^ T cells to the skin, thereby promoting the onset of acute GVHD.

### 3.9 CXCR1 and CXCR2

CXCL8, also known as interleukin-8 (IL-8), represents a pivotal molecule that triggers and activates neutrophils in response to tissue damage or infection (82). Upon binding to its receptors-CXCR1 and CXCR2, CXCL8 governs the activation and chemotaxis of neutrophils (83). Additionally, CXCL8 intensifies cellular apoptosis and oxidative stress via the activation of the NF-κB pathway, thereby propagating the development of inflammation (84, 85).

### 3.10 CXCR3

The IFNγ-IFNγR signaling pathway participates in regulating the directional migration of alloreactive T cells to target organs by upregulating the expression of the key chemokine receptor CXCR3 (86). Studies have shown that CXCR3 knockout in murine models significantly alters the migration pattern of T cells, causing them to preferentially migrate to the spleen rather than the gastrointestinal tract. This alteration markedly reduces the incidence of acute GVHD while preserving the necessary graft-versus-host response (GVL) (86). CXCL10, as a specific ligand for CXCR3, is one of the important biomarkers for acute GVHD (87, 88). TNF-α and IFN-γ secreted by Th1 cells promote the release of CXCL10 from parenchymal cells, acting on Th1 cells and forming a positive feedback loop, thereby exacerbating the activation of Th1 cells and tissue damage (89, 90).

### 3.11 CXCR4

Cytotoxic γδT cells migrate to the target organs of acute GVHD through CXCR4 mediation (91). In addition, CD4^+^ T cells, rather than CD8^+^ T cells, also depend on the migration of the CXCL12/CXCR4 axis to target organs in acute GVHD mice (91). Plerixafor, a small molecule inhibitor of CXCR4, has exhibited good efficacy in mobilizing hematopoietic stem cells from donors, accompanied by a lower incidence of acute GVHD and cytomegalovirus (CMV) viremia (92, 93). The number of CD56^bright^ regulatory NK cells (NKregs) in peripheral blood mobilized after plerixafor is significantly higher than that mobilized with granulocyte colony-stimulating factor (G-CSF) (94). This may be one of the mechanisms by which plerixafor exerts a protective effect in acute GVHD.

### 3.12 XCR1 and CX3CR1

XCL1 and its receptor XCR1 are involved in the pathological processes of various sterile inflammatory diseases (95–97). Activated CD8^+^ T cells and NK cells are the main sources of XCL1 (98). After binding to XCR1 on the surface of DC cells, XCL1 promotes the secretion of IL-12, thereby driving Th cells to differentiate towards Th1 and Th17 subsets (99). The high expression of CX3CR1 on the surface of CD8^+^T cells and the overexpression of its ligand CX3CL1 in the intestinal mucosa of patients with acute GVHD suggest the critical role of the CX3CR1/CX3CL1 axis in intestinal infiltration and damage during acute GVHD (100). Blocking CX3CL1 can effectively diminish the infiltration of alloreactive CD8^+^T cells in the intestine and alleviate the apoptosis of intestinal crypt cells, providing a new strategy for the treatment of intestinal damage in acute GVHD (101).

These research findings not only provide a new perspective for understanding the complex mechanisms of acute GVHD but also offer a theoretical basis for clinical intervention to alleviate acute GVHD symptoms by regulating the interaction between chemokine receptors and their respective ligands (**Table 1**).

**Table 1:** Chemokine-mediated T cells infiltration of target organs in acute GVHD.

Receptors	Chemokines	Primary target T cell	Infiltration organs	Ref.
CCR1	CCL5	Th cells, Tc cells	Liver and intestines	(56, 82)
CCR2	CCL2	CD8^+^ T cells	Liver, intestines, and oral	(70)
CCR4	CCL17, CCL22	CD8^+^ T cells, Tregs cells	Skin	(2)
CCR5	CCL3, CCL4, and CCL5	CD8^+^ T cells, Treg cells	Liver and intestines	(6, 23, 93, 102)
CCR6	CCL20	CD4^+^ T cells	Skin and intestines	(76, 90)
CCR7	CCL19, CCL21	Treg cells	Lymph nodes	(80)
CCR8	CCL8	Treg cells	Lymph nodes and intestines	(7)
CCR9	CCL25	Treg cells	Intestines	(51, 60, 71)
CCR10	CCL27	CD4^+^ T cells	Skin	(95)
CXCR3	CXCL10	Th1 cells	Spleen	(3, 12, 24, 31, 63)
CXCR4	CXCL12	CD4^+^ T cells, γδT cells	Liver and skin	(13)
CX3CR1	CX3CL1	CD8^+^T cells	Liver and intestines	(11, 61)

## 4 Clinical perspectives

Chemokines play a crucial role in the pathogenesis of acute GVHD and exhibit distinct expression patterns in the different target organs. Specifically, the upregulation of CXCL9, CXCL10, and CXCL11 in the skin of patients with acute GVHD promotes the recruitment of eosinophils and T cells to the sites of injury (102, 103). Similarly, elevated levels of CCL2, CCL3, and CCL5 in the liver and intestines of acute GVHD mice mediate the infiltration of neutrophils and activated T cells (104, 105). The differential expression of chemokines in the target tissues of acute GVHD suggests that they may serve as biomarkers for disease prediction and diagnosis.

Early detection of serum levels of CCL23 and CXCL9 in patients undergoing HSCT can effectively predict the risk of developing acute GVHD (106, 107). The significant increase in the levels of CXCL9 and CXCL10 in patients’ plasma can serve as effective diagnostic biomarkers for acute GVHD (103, 106, 108). Notably, reduced levels of CXCL8 in plasma on day +7 were associated with grade II–IV acute GVHD (109). However, pediatric patients displaying elevated levels of CXCL8 had a reduced risk of developing chronic GVHD, as opposed to acute GVHD, in comparison to patients who demonstrated lower levels of CXCL8 (110). Patients with skin GVHD who were treated with calcipotriene showed a significant decrease in skin CXCL10 levels (111). Furthermore, in acute GVHD mice treated with JAK inhibitors, a significant decrease in CXCL10 levels was observed, indicating that changes in chemokine levels can reflect the response of acute GVHD to effective therapy (112). Bogunia-Kubik et al. showed that the CCR5 Delta32 allele is an independent protective factor for patients developing acute GVHD, and the protective effect is more pronounced when the donor also carries this allele (113). Heightened levels of CCR5 indicated an increased susceptibility to systemic inflammation, thereby predisposing patients to multiple complications associated with transplantation. Furthermore, elevated CCR5 expression levels prior to transplantation were associated with unfavorable clinical outcomes (114).

In animal experiments, treatment with anti-CCR5 antibodies effectively reduced the infiltration of CCR5^+^CD8^+^ T lymphocytes in the liver, thereby alleviating liver damage caused by acute GVHD (115). In addition, the small molecule CCR5 antagonist Maraviroc, reduced the incidence of liver GVHD by blocking CCR5 and affecting T cell function (116). It is noteworthy that some patients failed to achieve complete blockade of CCR5 despite the administration of maraviroc. Inadequate CCR5 blockade was linked to a heightened risk of severe GVHD-related mortality and non-relapse mortality (114).

The addition of Maraviroc to the standard GVHD prevention regimen significantly reduces the incidence of acute gastrointestinal GVHD in patients without increasing the risk of disease recurrence (108). It is worth noting that although there was a slight increase of CCR5 expression in peripheral blood T cells on d +30 after transplantation, T cell activation was inhibited, which did not augment the risk of infection in patients after transplantation (108). Maraviroc-induced adverse effects were not observed in a study that investigated the efficacy and safety of Maraviroc in pediatric patients receiving HSCT (117). A phase II study was conducted to validate the efficacy of maraviroc administered from day -3 to day +30 post-HSCT for preventing acute GVHD in children (118). Although hepatotoxicity limited the use of Maraviroc, it has potential for the prevention of acute gastrointestinal GVHD (118). Furthermore, a phase 2 clinical trial reported that extending the use of Maraviroc to d +90 days showed a favorable preventive effect on both acute and chronic GVHD, significantly improving long-term survival (119). Varona et al. highlighted the key role of CCR6 in promoting the recruitment of alloreactive CD4^+^ T cells to acute GVHD target organs (72). The experiment showed that recipient mice receiving transplants from CCR6-deficient donors experienced delayed onset and relatively mild symptoms of acute GVHD, suggesting that CCR6 may be a potential chemokine receptor target for the prevention and treatment of acute GVHD (72). Additionally, in a xenograft experiment using CCR2 gene knockout donors, the infiltration of CCR2^−/−^ CD8^+^ T cells in the intestines and livers of recipient mice decreased, leading to reduced pathological damage in target organs and an overall decrease in the incidence and mortality of acute GVHD, while retaining the intact GVL (35). Furthermore, in a mouse GVHD model, He et al. found that anti-CXCR3 antibodies could reduce the infiltration of alloreactive CD8^+^ T cells into acute GVHD target organs (120). When acute GVHD mice were simultaneously treated with CCR5 and CXCR3 antagonists, the polarization of T cells towards Th1 and Tc1 was inhibited, and the generation of Treg was induced (121). Compared to the use of CCR5 or CXCR3 antagonists alone, the combined blockade of these two chemokine receptor antagonists more effectively reduced the incidence of acute GVHD and alleviated the clinical manifestations of acute GVHD in mice (121). This finding provides a potential novel approach for the prophylaxis and treatment of acute GVHD by combining different chemokine receptor antagonists.

## 5 Summary

In recent years, the role of chemokines and their receptors in the diagnosis and treatment of acute GVHD has been a hot topic of research. Chemokines play a crucial role in regulating immune cell differentiation, function, and migration, and both preclinical and clinical studies have demonstrated the involvement of chemokines in the initiation and progression of acute GVHD.

Despite the abundance of preclinical investigations pertaining to various chemokines, there is a profound lack of clinical studies, which may be due to the intricate nature of chemokines and the inherent challenges in their development as viable therapeutic agents. Given the complex pathogenesis of acute GVHD, monoclonal antibodies or antagonists targeting a single chemokine may not achieve optimal prevention of acute GVHD. Therefore, a combination of multiple chemokines or other preventive drugs for acute GVHD may offer greater benefits to patients. In patients afflicted with acute GVHD, the presence of concurrent comorbidities such as infections further complicates the chemokine axis involved, potentially influencing other molecular pathways or immune responses, and yielding unforeseen off-target effects. Moreover, the conundrum of chemokine redundancy remains unresolved, posing obstacles to targeted therapies. A solitary chemokine can bind to multiple receptors or conversely, multiple chemokines can activate a single receptor, thereby exacerbating the precision and efficacy hurdles in targeted therapies. Although in mouse experiments, blocking chemokines alleviates acute GVHD while preserving the GVL effect with negligible impact on the hematopoietic and immune systems, further research in human populations is necessary to evaluate its efficacy and safety. In conclusion, we anticipate more reports on the role of chemokine axes in the pathogenesis of acute GVHD to reduce transplant-related mortality and improve the prognosis of patients receiving allo-HSCT.
